# Supplementary material for: NucleiSky enables cross-scale multimodal registration of microscopy data using nuclei constellations
Source: J Cell Sci. 2026 Oct 14;139(21):jcs265152. doi: 10.1242/jcs.265152 (PMC13741949; doi:10.1242/jcs.265152)
Supplement: Supplementary file 1 [file joces-139-265152-s1.pdf]

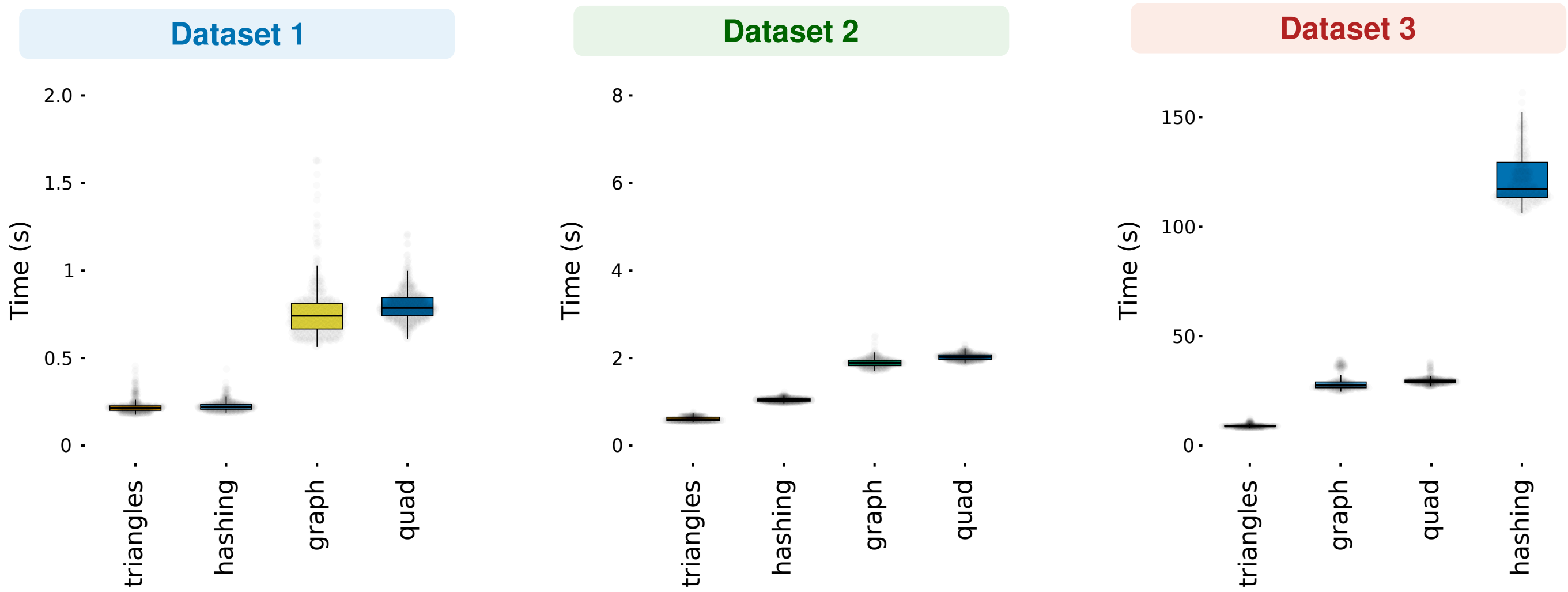

Fig. S1. Box plots show the distribution of execution times for each matcher to achieve a valid match across Datasets 1-3. To focus on true-positive localisation time, failed matching attempts are excluded from this analysis. In each dataset panel, the algorithms are ordered from left to right by increasing median execution time (fastest to slowest).

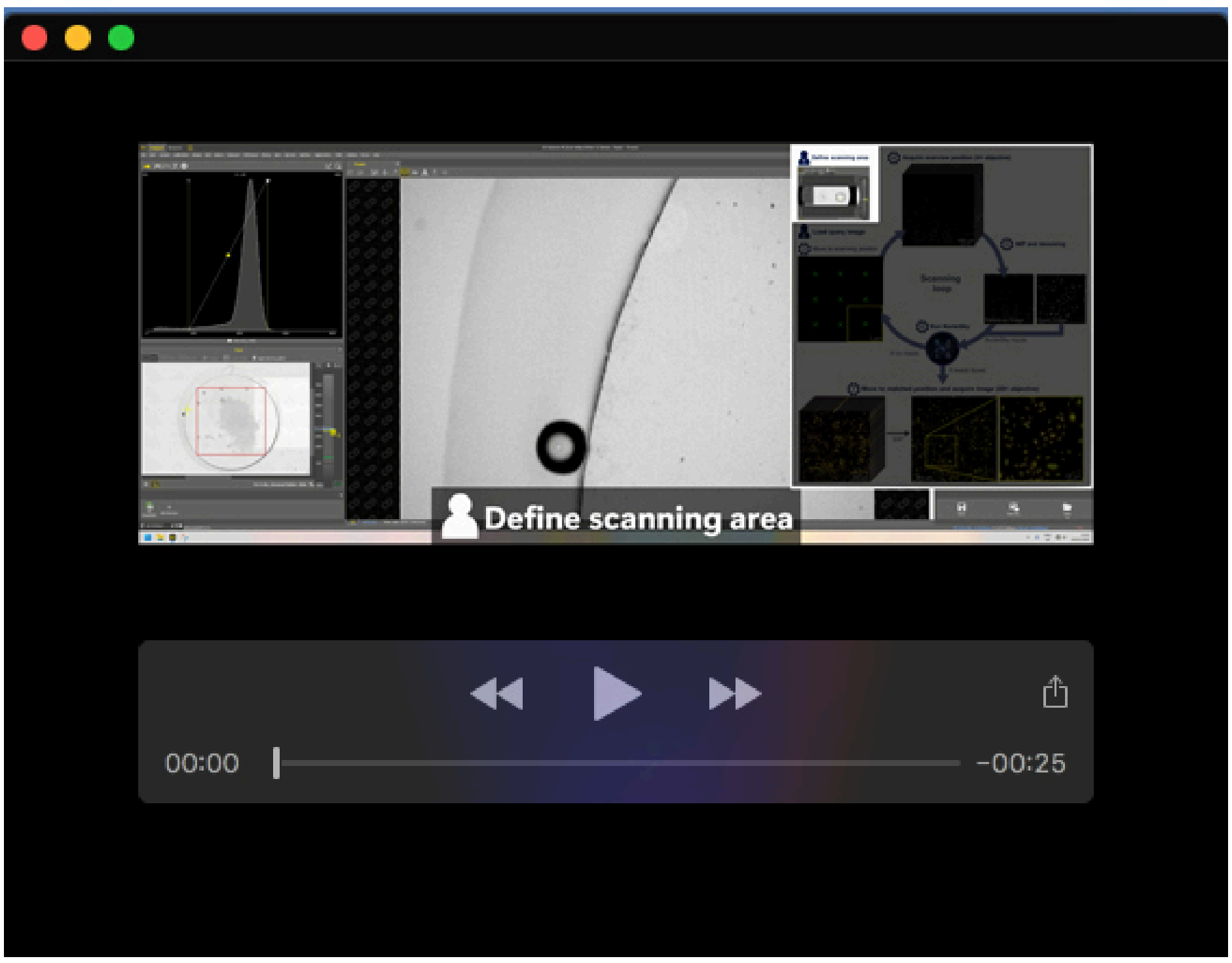

Movie 1. Smart microscopy relocalisation workflow in NIS-Elements AR.
Screen recording of the smart microscopy relocalisation workflow implemented using JOBS in NIS-Elements AR software, shown alongside Figure 5 to highlight the corresponding workflow step. The workflow aims to locate a query field-of-view image acquired on a different microscope than the microscope shown in the video. The video covers the complete process, including manual configuration of the acquisition area and input parameters, automated slide scanning, execution of NucleiSky to compare the query and reference images, and automated final image acquisition.

Journal of Cell Science • Supplementary information
