# Supplementary material for: NucleiSky enables cross-scale multimodal registration of microscopy data using nuclei constellations
Source: J Cell Sci. 2026 Oct 14;139(21):jcs265152. doi: 10.1242/jcs.265152 (PMC13741949; doi:10.1242/jcs.265152)
Supplement: Supplementary file 2 [file joces-139-265152_review_history.pdf]

Journal of Cell Science

NucleiSky enables cross-scale multimodal registration of microscopy data using nuclei constellations

Iván Hidalgo-Cenalmor, Adán Olguín-Olguín, Carolina Prieto, Johannes Kumra Ahnlide, Pontus Nordenfelt, Ricardo Henriques, Mario Del Rosario and Guillaume Jacquemet
DOI: 10.1242/jcs.265152

Editor: Francesca Bottanelli

Review timeline

Original submission:	29 June 2026
Editorial decision:	4 August 2026
First revision received:	5 August 2026
Accepted:	9 August 2026

Original submission

First decision letter

MS ID#: jcs.265152

MS TITLE: NucleiSky enables cross-scale multimodal registration of microscopy data using nuclei constellations

AUTHORS: Iván Hidalgo Cenalmor; Adán Olguín-Olguín; Carolina Prieto; Johannes Kumra Ahnlide; Pontus Nordenfelt; Ricardo Henriques; Mario Del Rosario; Guillaume Jacquemet
ARTICLE TYPE: Tools and Resources

Dear Guillaume,

We have now reached a decision on the above manuscript.

As you will see, the reviewers gave favourable reports and only raised minor concerns that will require amendments to your manuscript. I would be happy to accept your paper after you address all the reviewers points.

Reviewer 1

This manuscript by Cenalmor et al. describes a cross-scale multimodal image registration tool, NucleiSky. The fundamental idea is to leverage the principles of blind astrometry behind astronomical mapping. Image registration is become increasing important as biologists seek to address questions that span larger length scales and across more than one microscopy modality. NucleiSky, is designed to accomplish this and is offered as an open-source Python package.

I want to congratulate the authors for a well written manuscript with easy readability that is suitable for the intended biologists target audience. The authors have performed careful, well-considered and thorough validation on various aspects of the tool performance. This is the major strength of the paper, which I recommend acceptance following some minor changes.

Minor comments:

© 2026. Published by The Company of Biologists under the terms of the Creative Commons Attribution License (https://creativecommons.org/licenses/by/4.0/).

1. Table 1 reports the estimated landmark thresholds for 90% localization probability across the different matcher algorithms for each example dataset, it would be much easier to understand if they listed a summary of each dataset in that same table i.e., instead of just "Dataset 1" having the column entry be "Dataset 1: Intensity thresholded DAPI-stained endothelial monolayers," "Dataset 2: DAPI-stained endothelial monolayers segmented by Cellpose-SAM," "Dataset 3: H&E stained mouse colon section," etc. This would improve the readability rather forcing the readers to go back through the section to remember what each dataset number represents.

2. In the smart microscopy section, the MIP choice is fine for speed and convenience. However, the limitation of that approach can be addressed especially for densely packed nuclei this won't work well.

3. The authors only applied two geometric matchers (rather than all 4 they try in 2D), it is unclear why they only chose two. Perhaps the reasons can be more clearly articulated.

4. The section on live-to-fixed needs clarity. The problem it is trying to solve is not immediately clear until repeated reading. The main issue this tool seeks to tackle is quite important, namely, a biologist may be following live cellular event and need to follow up with a high-resolution immunofluorescence study afterward. It is usually a challenge to relocate the event site. The live-to-fixed feature fixes this. Yet, the original experimental challenge is never properly framed at the beginning of this section.

5. The authors also mentioned that many biologists rely on microfluidics devices to accomplish this task. Yet, fluidic devices also obviate the need for NucleiSky and switching of microscopes. Perhaps the authors can strengthen their argument in this case.

Other than these points, this is a very good paper describing a nifty tool that many readers will find extremely useful. The comments here are sufficiently minor that I do not need to read the revised manuscript again.

Teng-Leong Chew

Reviewer 2

SUMMARY OF THE ADVANCE MADE IN THIS PAPER AND ITS POTENTIAL SIGNIFICANCE TO THE FIELD

The manuscript presents a new method and a software for the registration of images, based on the spatial arrangement of nuclei or other structures. The method is inspired by the matching of views of stars onto maps in astronomy, but adapted to microscopy images in biology. Morphology features of the individual nuclei can be added in addition to the constellation features and the method is extended to 3D images. The transformations that can be handled are translations, rotations and uniform scaling. The authors propose 4 matching engines, that calculate and compare features in different ways, for the 2D case, and 2 for the 3D case. The software can automatically decide which matcher is best suited in a given situation. The benchmarking shows that the method can be quite robust with respect to segmentation errors.

This registration tool based on the spatial configurations of structures is a welcome addition to the usual methods. Automatic landmark extraction and correlation based methods will fail when the images are very different from each other due to different imaging modalities while the constellations of nuclei will still remain the same. This work will extend the possibility of fully automated registration of images to cases that could not be handled before. The open source software comes with a well designed API and can easily be integrated into python platforms like napari. The widget based notebooks provide an easy access without the need for programming. The software also allows to apply the calculated transformations to other channels. For these reasons a widespread adoption of the software within the community is to be expected if the software will be maintained in the future.

SUGGESTIONS TO AUTHORS

© 2026. Published by The Company of Biologists under the terms of the Creative Commons Attribution License (https://creativecommons.org/licenses/by/4.0/).

Major comments

I have no major comments. I recommend the manuscript for publication in its current form. Congratulations for this excellent work and thank you for providing the software with a reasonable API separately from the GUI version.

Minor comments

1. You have chosen to use the pyramid and geometric hashing matchers for 3D but you do not provide any arguments for your choice. They were probably the easiest to adapt to 3D, but wouldn't a generalization of the others be also possible? Are there any arguments why they wouldn't perform well in 3D?

2. You make the point, that an advantage of your method is, that it can be used for multimodal image registration. Yet the only example you provide outsources the problem, by transforming the images from one mode to the other beforehand. A more direct multimodal example would have been interesting. However I do not consider this as absolutely necessary, since it is sufficiently clear under which conditions this method works.

First revision

Author response to reviewers' comments

Reviewer 1: This manuscript by Cenalmor et al. describes a cross-scale multimodal image registration tool, NucleiSky. The fundamental idea is to leverage the principles of blind astrometry behind astronomical mapping. Image registration is become increasing important as biologists seek to address questions that span larger length scales and across more than one microscopy modality. NucleiSky, is designed to accomplish this and is offered as an open-source Python package.

I want to congratulate the authors for a well written manuscript with easy readability that is suitable for the intended biologists target audience. The authors have performed careful, well-considered and thorough validation on various aspects of the tool performance. This is the major strength of the paper, which I recommend acceptance following some minor changes.

Authors: We thank the reviewer for the positive comments and useful suggestions and for supporting the publication of our work.

Minor comments:

1. Table 1 reports the estimated landmark thresholds for 90% localization probability across the different matcher algorithms for each example dataset, it would be much easier to understand if they listed a summary of each dataset in that same table i.e., instead of just "Dataset 1" having the column entry be "Dataset 1: Intensity thresholded DAPI-stained endothelial monolayers," "Dataset 2: DAPI-stained endothelial monolayers segmented by Cellpose-SAM," "Dataset 3: H&E stained mouse colon section," etc. This would improve the readability rather forcing the readers to go back through the section to remember what each dataset number represents.

Authors: Excellent point. We have now implemented this suggestion.

2. In the smart microscopy section, the MIP choice is fine for speed and convenience. However, the limitation of that approach can be addressed especially for densely packed nuclei this won't work well.

Authors: This is true. We have now added a sentence to clarify this point.

3. The authors only applied two geometric matchers (rather than all 4 they try in 2D), it is

© 2026. Published by The Company of Biologists under the terms of the Creative Commons Attribution License (https://creativecommons.org/licenses/by/4.0/).

unclear why they only chose two. Perhaps the reasons can be more clearly articulated.

Authors: We thank the reviewer for this comment and agree that the rationale for selecting the two 3D matchers was not explained. The pyramid and geometric hashing matchers were selected to represent two complementary geometric-matching families that had both shown useful properties in 2D.

First, the pyramid matcher is the natural volumetric counterpart of the local triangle/quad family. In 2D, local configurations are represented using triangles or four-point quads; in 3D, the corresponding non-coplanar local primitive is a four-point tetrahedron. The pyramid matcher therefore generalises triangle-based simplex matching to 3D while also capturing the four-point local geometry represented by the 2D quad matcher. Consequently, separate 3D triangle and quad implementations would not provide the same distinction as they do in 2D.

Second, we implemented 3D geometric hashing because it provides a fundamentally different and complementary candidate-generation strategy. Rather than matching local descriptors directly, it constructs a three-dimensional coordinate frame from anchor landmarks and uses indexed retrieval and voting to identify compatible configurations. This approach was particularly attractive because the 2D hashing matcher was particularly tolerant of spurious detections.

We did not prioritise a 3D graph matcher because, in the 2D benchmarks, it was consistently less sample-efficient than the quad and triangle matchers. In addition, extending it to arbitrary 3D rotations would require a new rotation-invariant graph descriptor rather than a direct adaptation of the 2D method. We have revised the Results to clarify these selection criteria.

4. The section on live-to-fixed needs clarity. The problem it is trying to solve is not immediately clear until repeated reading. The main issue this tool seeks to tackle is quite important, namely, a biologist may be following live cellular event and need to follow up with a high-resolution immunofluorescence study afterward. It is usually a challenge to relocate the event site. The live-to-fixed feature fixes this. Yet, the original experimental challenge is never properly framed at the beginning of this section.

Authors: We thank the reviewer for this suggestion. We have now added a sentence to better introduce the section.

5. The authors also mentioned that many biologists rely on microfluidics devices to accomplish this task. Yet, fluidic devices also obviate the need for NucleiSky and switching of microscopes. Perhaps the authors can strengthen their argument in this case.

Authors: Excellent point; we have added a sentence on this topic in the discussion.

Other than these points, this is a very good paper describing a nifty tool that many readers will find extremely useful. The comments here are sufficiently minor that I do not need to read the revised manuscript again.

Teng-Leong Chew

Reviewer 2: SUMMARY OF THE ADVANCE MADE IN THIS PAPER AND ITS POTENTIAL SIGNIFICANCE TO THE FIELD

The manuscript presents a new method and a software for the registration of images, based on the spatial arrangement of nuclei or other structures. The method is inspired by the matching of views of stars onto maps in astronomy, but adapted to microscopy images in biology. Morphology features of the individual nuclei can be added in addition to the constellation features and the method is extended to 3D images. The transformations that can be handled are translations, rotations and uniform scaling. The authors propose 4 matching engines, that calculate and compare features in different ways, for the 2D case, and 2 for the 3D case. The software can automatically decide which matcher is best suited in a given situation. The benchmarking shows that the method can be quite robust with respect to segmentation errors.

© 2026. Published by The Company of Biologists under the terms of the Creative Commons Attribution License (https://creativecommons.org/licenses/by/4.0/).

This registration tool based on the spatial configurations of structures is a welcome addition to the usual methods. Automatic landmark extraction and correlation based methods will fail when the images are very different from each other due to different imaging modalities while the constellations of nuclei will still remain the same. This work will extend the possibility of fully automated registration of images to cases that could not be handled before. The open source software comes with a well designed API and can easily be integrated into python platforms like napari. The widget based notebooks provide an easy access without the need for programming. The software also allows to apply the calculated transformations to other channels. For these reasons a widespread adoption of the software within the community is to be expected if the software will be maintained in the future.

We thank the reviewer for taking the time to review our manuscript.

SUGGESTIONS TO AUTHORS

Major comments

I have no major comments. I recommend the manuscript for publication in its current form. Congratulations for this excellent work and thank you for providing the software with a reasonable API separately from the GUI version.

We thank the reviewer for supporting the publication of our work.

Minor comments

1. You have chosen to use the pyramid and geometric hashing matchers for 3D but you do not provide any arguments for your choice. They were probably the easiest to adapt to 3D, but wouldn't a generalization of the others be also possible? Are there any arguments why they wouldn't perform well in 3D?

Authors: As also noted in our response to Reviewer 1, other 3D extensions are possible, and we do not assume that they would perform poorly. Our aim was to establish the feasibility of 3D constellation-based localisation rather than exhaustively compare all potential 3D matchers. We selected two complementary methods with direct volumetric formulations: the pyramid matcher generalises the local triangle/quad strategy to tetrahedra, whereas geometric hashing uses 3D anchor frames and indexed retrieval. This choice also reflects their complementary behaviour in 2D, where four-point matching was most sample-efficient, and hashing was most tolerant of spurious detections.

We did not prioritise a 3D graph matcher because it was less sample-efficient in our 2D benchmarks. Its extension would also require implementing a new rotation-invariant 3D descriptor and separate validation. Other 3D graph or higher-order formulations remain possible, but their relative performance is currently unknown. We have clarified these considerations in the revised Results.

2. You make the point, that an advantage of your method is, that it can be used for multimodal image registration. Yet the only example you provide outsources the problem, by transforming the images from one mode to the other beforehand. A more direct multimodal example would have been interesting. However I do not consider this as absolutely necessary, since it is sufficiently clear under which conditions this method works.

Authors: We agree that the live-to-fixed example does not constitute fully direct multimodal registration at the raw-image level, because artificial labelling is used to generate comparable nuclear landmarks. However, this step does not produce exact correspondence: indeed, the fake nuclei labels contain positional errors, missed nuclei and spurious detections relative to the fixed fluorescence image. The experiment therefore also tests whether NucleiSky can recover an alignment from imperfect landmarks inferred across modalities. Nevertheless, direct registration

© 2026. Published by The Company of Biologists under the terms of the Creative Commons Attribution License (https://creativecommons.org/licenses/by/4.0/).

using landmarks independently detected in substantially different modalities would provide valuable additional validation and remains an important direction for future work.

Second decision letter

MS ID#: jcs.265152R1

MS Title: NucleiSky enables cross-scale multimodal registration of microscopy data using nuclei constellations

Authors: Iván Hidalgo Cenalmor; Adán Olguín-Olguín; Carolina Prieto; Johannes Kumra Ahnlide; Pontus Nordenfelt; Ricardo Henriques; Mario Del Rosario; Guillaume Jacquemet
Article Type: Tools and Resources

Dear Guillaume,

I am happy to tell you that your manuscript has been accepted for publication in Journal of Cell Science, pending standard publication integrity checks.

© 2026. Published by The Company of Biologists under the terms of the Creative Commons Attribution License (https://creativecommons.org/licenses/by/4.0/).
